# Effects of washing with boric acid solutions on residual boric acid content, microbiological load, and quality of fresh-cut spinach

**DOI:** 10.1016/j.heliyon.2024.e31974

**Published:** 2024-05-25

**Authors:** Bahar Demircan, Yakup Sedat Velioglu, Angelo Maria Giuffrè

**Affiliations:** a Department of Food Engineering, Faculty of Engineering, Ankara University, Ankara, Türkiye; b Department of AGRARIA, Università degli Studi Mediterranea, di Reggio Calabria, Italy

**Keywords:** Spinach, Fresh-cut, Boric acid, Disinfectant, Shelf life, Food safety

## Abstract

Insufficient disinfection of fresh-cut spinach poses significant health risks, along with potential issues like odor, color changes, and softening during short-term storage. To address these challenges, boric acid solutions were explored as an alternative to chlorine washes, which are known to produce toxic compounds. Among various concentrations, 1 % boric acid exhibited the most effective microbial inactivation, leading to substantial reductions in total mesophilic aerobic bacteria, total yeast and mold, and *Enterobacteriaceae* counts, with reductions of 1.64, 1.38, and 1.77 logs, respectively. Additionally, washing spinach leaves with this solution for 1 min maintained quality parameters, with enhanced antioxidant activity (55.26 mg kg^−1^ Trolox equivalent), increased total phenolic content (1214.06 mg kg^−1^ gallic acid equivalent), retention of chlorophyll *a* (839.16 mg kg^−1^), chlorophyll *b* (539.61 mg kg^−1^) and ascorbic acid content (264.72 mg kg^−1^). Mechanical properties such as puncture strength (1.81 N) and puncture distance (52.78 mm) also showed favorable outcomes, alongside optimal moisture content at 89.81 %. Notably, residual boric acid content was lowest in spinach leaves (1252.49 mg kg^−1^) and highest in the wash water (53.88 mg kg^−1^) after treatment. Scanning electron microscopy images demonstrated maintained tissue integrity, while Hunter Lab readings indicated minimal color changes post-washing. Additionally, sensory evaluations and various physicochemical analyses further supported the efficacy of boric acid washing. Consequently, washing spinach leaves with a 1 % boric acid solution for 1 min yielded favorable results across multiple quality parameters. These findings suggest the potential of boric acid as a safe and effective alternative disinfectant in the fresh-cut produce industry, highlighting its practical implications for food safety and quality. Future research should focus on exploring long-term effects and optimizing washing protocols for broader applications.

## Highlights

• The washing process with boric acid has preserved the quality of fresh-cut spinach.
• Washing with boric acid has been evaluated as an effective disinfectant.
• Short-term washing with boric acid is the most effective practice.
• Consumers have accepted fresh-cut spinach washed with boric acid.
• Washing with boric acid has increased the shelf life of fresh-cut spinach.

## Graphical abstract

**Figure:**
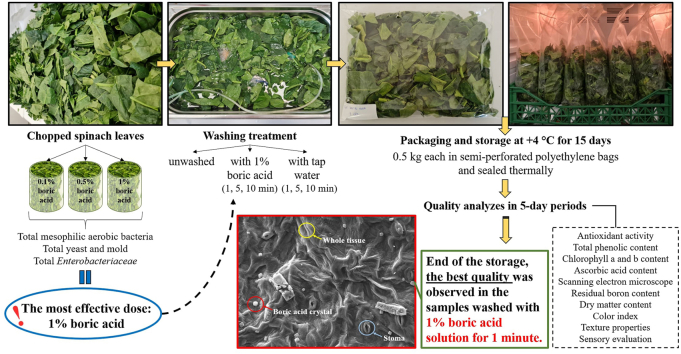

## 1 Introduction

Leafy green vegetables are essential to a healthy diet as they are a rich source of important vitamins, dietary fibre, minerals, and phytonutrients. Their fresh-cut, ready-to-eat form is increasingly popular in line with changing consumer habits. Ready-to-eat, leafy green vegetables retain most of their existing microflora despite minimal processing, posing a potential food safety issue. In 2008, FAO-WHO designated leafy green vegetables as a top priority in terms of fresh produce safety from a global perspective [1]. Spinach is a significant leafy vegetable because of its rich nutritional content, including carotenoids (β-carotene and lutein), vitamin C, vitamin E, vitamin K, magnesium, and flavonoids. Today, with the changing consumer preferences, it is becoming more and more common to buy spinach as fresh-cut (washed, chopped, ready for consumption). However, fresh-cut spinach may encounter quality issues during storage, such as a strong odor, deterioration, discoloration, and texture softening [2]. Generally, the characteristic microflora in fresh vegetables consists mainly of gram-negative bacteria, which are the primary contaminants responsible for vegetable spoilage. The emergence of bacteria from the *Enterobacteriaceae* group suggests insufficient disinfection during the post-harvest and processing stages of vegetables. Consequently, if fresh-cut spinach does not undergo a thorough and effective cleaning process, it may present a significant food safety hazard [3].

Safe production methods and proper disinfection or decontamination procedures are essential steps in ensuring the food safety of fresh-cut vegetables. Various disinfection methods have been developed, such as chlorine, chlorine dioxide, bromine, iodine, trisodium phosphate, hydrogen peroxide, ozone, and some acids. Among these, chlorine is the most commonly used disinfectant, but the use of chlorine for washing is decreasing [4]. There is a growing need to develop alternative technologies because of increasing awareness of the adverse effects of chlorine, its by-products on health, and its harmful environmental effects [5,6]. In this context, the development of new decontamination methods should consider the impact of novel approaches on industrial practicality, organoleptic properties and shelf life factors. In medicine, in the field of sterilization and cleaning, in agriculture, and various industrial areas (glass, ceramic, metal, cosmetics, etc.), boric acid (BA) and its salts are used. However, their using in the food field could be more extensive [7]. BA, a key by-product of boron production, is a Lewis acid of boron with the chemical formula H_3_BO_3_ and is available in the form of water-soluble, colorless crystals or white powder [8]. The biological half-life of BA is shorter than one day, and it is classified as a non-toxic substance [9]. According to the EFSA report [10], BA and sodium tetraborate do not raise concerns regarding genotoxicity, and an intake of 0.16 mg of boron per kilogram of body weight per day can be regarded as an acceptable daily intake (ADI). Similarly, Hadrup et al. [11] also reported that boron-containing compounds were not genotoxic nor carcinogenic in genotoxicity studies. Baskan et al. [12] stated that the toxic effect of boron is shallow, and the lowest lethal dose determined for BA is 640 mg kg body weight^−1^ in humans when taken orally. Borax and boric acid have been discovered to be used for preserving foods since ancient times, and the addition of borate has been recognized as one of the best methods for preserving fish, meat, and dairy products. The Panel on Food Additives and Nutrient Sources Added to Food (ANS) provided a scientific opinion evaluating the safety of boric acid (E284) and sodium tetraborate (borax) (E285) as food additives in the European Union, stating that the use of these additives for preservation purposes in sturgeon fish eggs (caviar) up to a concentration of 4 g of boric acid per kg is permitted [7]. Boric acid is regulated as a food additive in various countries to ensure consumer safety, and there are also various reports on this issue. In Indonesia, its use in food products is strictly banned by the Regulation of the Minister of Health. Likewise, Malaysia prohibits its addition during food processing due to health concerns. Internationally, regulatory bodies such as the FDA, USDA, EFSA and JECFA work towards establishing regulations for the safe use of food additives like boric acid. These regulations focus on assessing safety, authorization procedures, and labelling of food additives to ensure their safe use in food products. Furthermore, evaluation processes for food additives in countries such as Argentina, Australia, Brazil, Canada, China, the EU, Japan, New Zealand, and the US aim to guarantee the safety of foods containing additives like boric acid [[13], [14], [15], [16], [17]].

The primary target of BA is microbial membranes, which it can easily penetrate. The mechanism of action of BA involves inhibiting membrane proteins or intracellular enzymes, leading to dysfunction in metabolic pathways and a slowdown in microbial metabolic processes. This occurs by impeding the transport of essential nutrients. The antimicrobial effect of BA depends on the target microorganism, exposure time, and concentration, and these parameters are essential for a bactericidal or bacteriostatic effect on microorganisms [18,19]. The effect of boron components has been studied only against certain microorganisms in a prepared nutrient medium and limited numbers. In an in vivo study conducted by Qin et al. [20], the effectiveness of the boron potassium tetraborate form was evaluated for post-harvest control of *Botrytis cinerea* on grapes. Significant results were observed with a 1 % concentration after a 15-min dipping time. The in vitro study conducted by Qin et al. [21] demonstrated that the treatment of BA effectively suppressed the growth of *Penicillium expansum*. BA was utilized by Rolshausen and Gubler [22] to control *Eutypa dieback* disease in red wine grapes. Yildirim et al. [23] effectively hindered the growth and spore germination of *P. expansum*, the fungus responsible for apple blue mold, by applying of BA at concentrations ranging from 0.125 % to 2 %. Ilhan et al. [24] provided the MIC (minimum inhibitory concentration) and MBC (minimum bactericidal concentration) values of BA, which were determined as 1.93 mg mL^−1^ for *Listeria monocytogenes* and 3.80 mg mL^−1^ for *Staphylococcus aureus*. In their study, Lai et al. [25] investigated the effect of BA on the virulence of *P. expansum*, a prominent pathogen in pome fruits that causes severe health problems and acts as the primary producer of patulin. As a result, it was observed that BA solution significantly inhibited the in vitro growth of *P. expansum* and decreased patulin production. Zan et al. [26] determined the high antibacterial activity of BA solution in *Enterococcus faecalis* biofilms. Upon analyzing the available literature, the treatment of a BA solution demonstrates efficacy as an antimicrobial agent. While there is limited research [20,21] on the inhibitory effects of BA, no studies have specifically explored the impact of BA on the quality of ready-to-eat food products during their storage.

In light of the extensive research conducted on the antimicrobial properties of boron components, our research aims to fill a critical gap in the literature by exploring the impact of washing with boric acid, specifically on the quality of ready-to-eat food products during storage. While previous studies have investigated the effectiveness of boron components against various microorganisms, limited attention has been given to their potential effects on the sensory and physicochemical properties of fresh-cut produce. Therefore, our research seeks to elucidate the unique contribution of boric acid in maintaining the quality and safety of fresh-cut spinach, providing valuable insights into their using as alternative disinfection agents in the food industry. In this study, the microbial inactivation effectiveness of boric acid solutions at different concentrations (0.1 %–1 %) on fresh-cut spinach leaves was evaluated to determine the effective disinfection dose. Additionally, the quality characteristics of fresh-cut spinach subjected to different washing times (1, 5, and 10 min) with the solution at this dose were examined during storage at 4 °C for 15 days. Various analyses were conducted to determine quality parameters, including antioxidant activity, total phenolic content, chlorophyll levels, ascorbic acid content, mechanical properties, and moisture content. Scanning electron microscopy images were used to assess leaf tissue integrity. Furthermore, residual boron levels in both the product and the washing solution were determined during the use of boric acid for washing purposes. By addressing this important aspect, our study aims to pave the way for the development of sustainable and environmentally friendly approaches to food safety, ultimately benefiting both consumers and producers alike.

## 2 Materials and methods

### 2.1 Material

Matador variety spinach (*Spinacia oleracea* L.), a highly nutritious leafy green vegetable from the Chenopodiaceae family, was selected for this experiment. The spinach was sourced from a market in Ankara, Türkiye, in March and April 2022, when it is typically at its freshest and most readily available. Roots and stems were removed, and leaves were cut into 3–4 cm stripes with a stainless steel knife and divided into 0.5 kg portions. Washing treatments were done in a stainless steel tank. 5 L of washing solution was used with spinach: solution ratio 1: 10 (w/v). Washing efficiency was increased with air bubbles obtained from an aquarium pump and bubbler stone. To determine the adequate BA solution treatment time, samples were treated accordingly: Unwashed (UW), washed with tap water (TW), and the previously determined effective BA solutions for 1, 5, and 10 min (TW1, TW 5, TW 10 and BA1, BA 5, BA10), respectively. During washing, the water temperature was maintained at 20 °C. The washed samples were taken from the pool, and excess water was removed using a centrifugal-based vegetable dryer and left to dry on filter papers [27].

All the chemicals were purchased from Sigma, Merck, C. Erba, and Oxoid. All experiments were performed in three replications, and three parallel measurements were conducted within each replication. All results are expressed as standard deviation.

### 2.2 Determination of the effective boric acid concentration by microbiological analysis

Spinach samples were washed for 5 min with 0.1, 0.5, and 1 % w/v of BA solutions to determine the effect of the BA solution on the natural microflora and the effective BA concentration, as explained in the Material section. Then, total mesophilic aerobic bacteria count (TMAB), total yeast and mold count (TYM), and total *Enterobacteriaceae* (TE) counts were performed. The results were determined as “log CFU g^−1^ spinach” [28].

### 2.3 Storage of samples

The treated samples weighing 0.5 kg were placed into thick, transparent polyethylene bags (ISOLAB, Türkiye) with dimensions of 200x300 mm. These bags were manually perforated with holes of 6 mm in diameter to improve ventilation and are designed to withstand punctures and tears. Afterwards, the bags were thermally sealed. 7 groups of samples were stored in cooled incubators (Velp FTC 901, Italy) at a temperature of +4 ± 0.1 °C for 15 days. The analyses were performed at 5-day intervals.

### 2.4 Analysis of antioxidant activity

Homogenized spinach leaves (1.5 ± 0.001 g) were mixed in a Falcon tube with 25 mL methanol: water mixture (80: 20, v/v), shaken at 220 rpm on an orbital mixer (Biosan, OS-10, Lithuania) for 1 h and centrifuged at 27123 g for 10 min (Hettich zentrifugen-Universal 320R, Germany). The supernatant was filtered through a 0.45 μm PTFE filter (Millipore Millex-LCR, Hydrophilic, PTFE, Bedford, USA), and this filtrate was used for both antioxidant activity and total phenolic content analysis.

60 μL of the extract was vortexed with 1940 μL of methanolic DPPH (2,2-diphenyl-1-picrylhydrazyl) solution (6 × 10^−5^ M) and kept in the dark for 1 h. At the end of the period, the absorbance of the samples was measured using a spectrophotometer (Shimadzu, UV-1601, Japan) at 515 nm. Antioxidant activity analysis results were determined regarding mg kg^−1^ Trolox equivalent [27].

### 2.5 Analysis of total phenolic content

Filtered 20 μL of extract, 1580 μL of distilled water, and 100 μL of Folin-Ciocalteu reagent were mixed in a test tube and were kept in the dark for 3 min, and then 300 μL of sodium carbonate solution (20 %, w/v) was added, vortexed again, and kept in the dark for 2 h. At the end of the period, the absorbance values of the samples were read at 765 nm in the spectrophotometer. Results were given as mg kg^−1^ gallic acid equivalent (GAE) [29].

### 2.6 Analysis of chlorophyll *a* and *b*

The homogenized 1 g leaf sample was stirred with 100 mL of 99.8 % (v/v) methanol, filtered through coarse filter paper and centrifuged at 20230 *g* for 10 min. The supernatant was then filtered through a 0.45 μm PTFE filter and injected into high-pressure liquid chromatography (HPLC) (Shimadzu, Prominence series, Japan) under the same conditions as reported by Karaca and Velioglu [27]. The results were quantitatively calculated using standard curves for chlorophyll *a* and *b*.

### 2.7 Analysis of ascorbic acid

Ascorbic acid analysis was conducted following the method described by Reyes et al. [30]. Specifically, a homogenized sample weighing 4 ± 0.001 g was mixed with 24 mL of 3 % (v/v) citric acid solution. The mixture was then filtered through coarse filter paper and centrifuged at 22415 g for 10 min. The resulting supernatant was collected and passed through a C18 solid-phase extraction cartridge (Phenomenex, Strata C18-E, 55 μm-70A, Torrance, USA). Prior to sample injection into the HPLC system, the cartridge was conditioned with 3 mL methanol and 3 mL of distilled water, following the conditions described by Karaca and Velioglu [27]. Ascorbic acid concentrations were determined by calculating the values from a calibration curve prepared using an analytical standard of ascorbic acid.

### 2.8 Determination of residual boron content

The residual boron content was determined in the samples washed with BA and the remaining washing solutions. First, the relevant samples were dried in an oven at 75 °C until they reached a constant weight. Subsequently, they were burned in a porcelain crucible at 525 °C in a muffle furnace. To the resulting ash, 10 mL of 2 N HNO_3_ was added, and the mixture was heated on a hot plate until complete dissolution. The solution was then filtered through the Whatman No 42 filter paper and diluted to a final volume of 50 mL with deionized water. Boron concentrations were measured using the spectrophotometric Azomethine H method, with BA used as a standard [31].

### 2.9 Determination of moisture content

The moisture contents of the samples were determined gravimetrically by drying them in an oven at 105 ± 5 °C (Nuve, FN120, Turkiye) until a constant weight was reached for approximately 24 h [32]. To prevent errors that may occur due to water loss during storage, the values of chlorophyll, ascorbic acid, total phenolic content, and antioxidant activity were determined based on the dry matter amounts of the vegetables.

### 2.10 Color measurement

The L* (100 white, 0 black), a* (+red, -green), and b* (+yellow, -blue) values of the samples were measured using a colorimeter (Konica Minolta, CR-400, Japan) [33].

### 2.11 Scanning electron microscope (SEM) analysis

After homogenization, freeze-dried leaf samples (TOPT-10D Shaanxi, China) were coated with gold at a current of 25 mA under a vacuum of a 9 × 10^−2^ mbar (EMITECH, K550X, Kent, UK) for SEM imaging (ZEISS, EVO 40, Jena, Germany). Surface images were recorded at the Ankara University Nuclear Sciences Institute Electron Microscopy Unit using a ZEISS EVO 40 microscope at an acceleration voltage of 20 kV and 1000× magnification.

### 2.12 Texture analysis

The textural quality of spinach samples was evaluated using the testing parameters outlined by More et al. [34] on a texture analysis device (Stable Micro Systems, TA.XT Plus, Surrey, UK). The samples were securely positioned on the support equipment (HDP/FSR) and subjected to a puncture test using a cylindrical probe (P/5S), which recorded force curves based on the puncture force and distance during deformation. The maximum force recorded was considered the puncture strength (N), and the displacement at which the puncture occurred was measured as the puncture distance (mm).

### 2.13 Sensory analysis

The color, texture, odor, and general acceptance of the samples were assessed through a collective evaluation by 30 semi-trained panellists using a 5-point scale during each storage period. The color evaluation ranged from dark green (5) to a more yellowing point (1), while the texture assessment ranged from very firm and stiff leaf (5) to very loose and pale leaf (1). Odor was rated on a scale from no odor (5) to a strong malodor (1). General acceptability was judged based on an excellent or fresh appearance (5) to a non-marketable quality (1). Samples that received a score below 3 in any of these sensory characteristics were considered to have “unacceptable marketability” [35,36].

### 2.14 Statistical analysis

The data obtained from the stored sample groups were analyzed using a factorial analysis of variance (ANOVA), and the Duncan test was used for making comparisons. All statistical analyses were conducted using SPSS 22.0 software (SPSS Inc., Chicago, USA). Statistical significance was determined at a threshold of *P* < 0.05, indicating that differences with a p-value less than 0.05 were considered significant.

## 3 Results and discussion

### 3.1 Determination of the effective dose of the boric acid solution by microbiological analysis

**Fig. 1:**
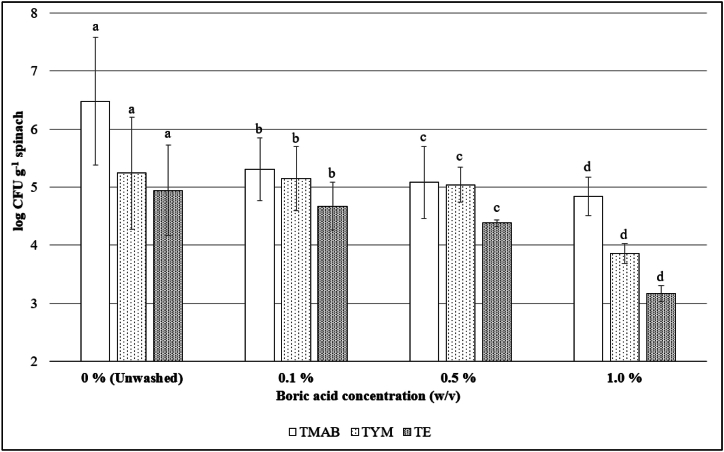
Effects of washing with various concentrations of boric acid solutions on total mesophilic aerobic bacteria (TMAB), total yeast and mold (TYM), and total *Enterobacteriaceae* (TE). Treatment time was 5 min in all. (^a^ Different letters used in the TMAB, TYM, and TE columns indicate significant differences (*P* < *0.05*) between each concentration according to the Duncan's test.).

In order to determine the optimal dosage of BA solution, spinach leaves were treated with solutions of different concentrations, followed by microbiological analysis targeting bacterial, mold, and yeast populations. In UW samples, TMAB, TYM, and TE were 6.48 ± 1.10, 5.24 ± 0.97, and 4.94 ± 0.78 log CFU g^−1^, respectively. In spinach washed with 0.1 %, 0.5 %, and 1 % of BA solutions, TMAB counts were determined as 5.31 ± 0.54, 5.08 ± 0.62, 4.84 ± 0.33; TYM counts of 5.15 ± 0.55, 5.04 ± 0.30, 3.86 ± 0.17, and TE counts of 4.67 ± 0.41, 4.38 ± 0.05, 3.17 ± 0.14 log CFU g^−1^, respectively. TMAB, TYM and TE decreased by 1.17, 0.09 and 0.27 log after washing with 0.1 % BA; 1.4, 0.2, 0.56 log after washing with 0.5 % BA; and 1.64, 1.38, 1.77 log after washing with 1 % BA, respectively. Statistical analysis revealed a significant difference among the samples, indicating a notable variation in the TMAB, TYM, and TE counts after washing with different concentrations of BA solutions (Fig. 1). The decreasing trend in microbial counts (TMAB, TYM, and TE) with increasing concentrations of BA solution suggests the effectiveness of BA in reducing microbial load on the spinach samples. The highest reductions in TMAB, TYM, and TE counts were observed after washing with the 1 % BA solution, demonstrating its strong antimicrobial effect. These findings highlight the potential of BA as an alternative disinfection agent in the fresh-cut industry for improving spinach's microbiological quality and safety.

The primary degradation mechanisms in fresh-cut leafy vegetables are tissue metabolism and microbial growth. Therefore, a disinfectant must first affect the natural microflora of the product and reduce the microbial load to non-harmful levels. Mainly, in fresh-cut spinach, even after minimal processing, the main microflora contains *Enterobacteriaceae*, and this group of microorganisms indicates inadequate disinfection. According to our results, washing the fresh-cut spinach with 0.1 %–1.0 % (w/v) BA solutions effectively inhibited the product's natural flora, and this concentration was used in the following experiments (Fig. 1). Reducing the pH is an effective way to control microorganisms' growth or growth rate. This is especially true for products with a natural pH in the 5 to 7 range where bacterial degradation is rapid [37]. Since the pH of the product became more acidic with increasing concentrations of BA solutions, 1 % BA was the most effective washing treatment. Similarly, in a study by Qin et al. [20], grapes were washed with the potassium tetraborate form of boron for post-harvest protection, and it was reported that the 1 % dose was the most effective concentration. Yildirim et al. [23] reported that the antimicrobial effect increased as the concentration of BA solutions increased, with a concentration range of 0.125 %–2 % in apples.

The main target of BA is microbial membranes, where it acts to slow down the microorganisms' metabolic processes [18]. The antimicrobial effect of BA varies depending on factors such as the target microorganism, exposure time, and concentration. These parameters are critical determinants for achieving either a bactericidal or bacteriostatic effect on microorganisms [19]. In the food industry, careful attention should be given to the form and concentration of boron (0.16 mg boron kg^−1^) used, as well as the composition and properties of the food product [12]. Additionally, when employing BA as a food additive, it is crucial to consider consumers’ daily intake levels for their health [10]. Our findings were consistent with the literature, demonstrating that BA exhibited greater efficacy than specific washing solutions commonly utilized in the fresh-cut industry. For instance, previous research conducted on fresh-cut spinach showed that tap water washing followed by packaging in polypropylene containers resulted in mesophilic bacteria, *Enterobacteriaceae*, and total yeast and mold counts of 7.2–7.9, 5.5–6.2, and 4.5–5.5 log CFU g^−1^, respectively [38].

Similarly, using peroxyacetic acid and hypochlorous acid solutions for washing spinach reduced the initial aerobic bacteria count from 6.5 to 6 CFU g^−1^ and from 6 to 5.5 CFU g^−1^, respectively [39]. Gu et al. [40] also reported a decrease in bacterial population from 6 to 4.7 log CFU g^−1^ after washing with chlorinated water. Consequently, our study underscores the superior microbial reduction achieved by BA in the processing of fresh-cut spinach.

### 3.2 Antioxidant activity and total phenolic content

Phenolic acids and flavonoid derivatives with antioxidant activity are essential groups of phytochemicals found in high amounts in leafy vegetables, and they must remain as stable as possible during storage. Mainly applied treatments, storage time, and conditions significantly affect retained phytochemical compounds on fresh-cut products [2]. Spinach has a very high antioxidant capacity due to its high total phenolic content, suggesting that regular consumption benefits health [38]. Increasing consumption of spinach has encouraged producing the fresh-cut form of this product. Current minimal processing procedures in the production of fresh-cut produce may affect the antioxidant phenolics and vitamin C content [41]. Although the losses of nutritional components are due to different factors, fresh-cut product processing also destroys phytochemical compounds. Antioxidant phytochemicals can degrade rapidly in fresh-cut products as they are exposed to light and oxygen by cutting tissues [2]. Antioxidant activity and total phenolic content decreased during storage in all sample groups. However, these contents were higher in samples treated with TW and BA compared to UW samples (Table 1). This decrease in polyphenolic compounds can be explained by the inability to make new syntheses due to the oxidation of these compounds and the ageing metabolism of the plant [42]. At the beginning and end of storage, the lowest antioxidant activity and total phenolics content were observed in the UW samples (47.65–40.21 mg kg^−1^ Trolox equivalent and 1197.27–1053.04 mg kg^−1^ GAE equivalent, respectively), and the highest in the BA1 samples (65.42–55.26 mg kg^−1^ Trolox equivalent and 1394.24–1214.06 mg kg^−1^ GAE equivalent, respectively). Similar to our results, the literature has reported that spinach leaves contain approximately 1000–1200 mg kg^−1^ of total flavonoids [43].

**Table 1:** Changes in some quality parameters of sample groups during storage periods.

	Sample^b^	Day 0	Day 5	Day 10	Day 15
**Moisture content (%)**	UW	89.19 ± 0.23^d.^	88.76 ± 0.05^b^	87.82 ± 0.16^c^	86.57 ± 0.01^a^
	TW1	90.23 ± 0.11^e^	88.77 ± 0.14^b^	88.36 ± 0.22^d^	87.36 ± 0.20^b^
	TW5	90.26 ± 0.97^f^	89.01 ± 0.23^d^	88.85 ± 0.17^e^	88.29 ± 0.14^c^
	TW10	90.71 ± 0.54^g^	90.59 ± 0.20^e^	90.07 ± 0.07^g^	88.81 ± 0.37^d^
	BA1	88.69 ± 0.08^c^	88.79 ± 0.54^c^	89.52 ± 0.41^f^	89.81 ± 0.41^g^
	BA5	87.31 ± 0.13^b^	87.60 ± 0.65^a^	87.73 ± 0.50^b^	89.33 ± 0.97^f^
	BA10	87.29 ± 0.74^a^	87.59 ± 0.10^a^	87.66 ± 0.08^a^	89.16 ± 0.65^e^
**Antioxidant activity (mg kg**^**−**^**^1^ Trolox eq)**	UW	47.65 ± 27.84^g^	47.20 ± 12.65^g^	46.51 ± 11.97^g^	40.21 ± 20.14^g^
	TW1	61.32 ± 19.98^b^	59.91 ± 11.97^b^	57.36 ± 10.87^b^	50.23 ± 18.54^b^
	TW5	54.65 ± 20.54^e^	50.42 ± 21.41^e^	49.71 ± 14.87^e^	48.24 ± 11.36^c^
	TW10	50.72 ± 21.47^f^	49.85 ± 17.56^f^	47.38 ± 13.87^f^	45.77 ± 20.32^f^
	BA1	65.42 ± 30.12^a^	60.28 ± 20.15^a^	58.74 ± 15.54^a^	55.26 ± 10.91^a^
	BA5	60.74 ± 35.65^c^	58.24 ± 15.50^c^	56.42 ± 21.52^c^	47.38 ± 17.32^d^
	BA10	58.48 ± 18.98^d^	56.47 ± 16.85^d^	50.84 ± 20.01^d^	46.10 ± 23.33^e^
**Total phenolic content (mg kg**^**−**^**^1^ GAE eq)**	UW	1197.27 ± 27.98^g^	1172.27 ± 26.54^g^	1164.09 ± 26.98^g^	1053.04 ± 25.21^g^
	TW1	1384.36 ± 21.22^b^	1370.35 ± 30.21^b^	1267.04 ± 21.65^c^	1208.67 ± 19.87^b^
	TW5	1264.62 ± 37.52^e^	1254.38 ± 36.23^e^	1217.17 ± 27.65^d^	1193.64 ± 34.12^e^
	TW10	1204.31 ± 30.12^f^	1190.91 ± 20.54^f^	1182.37 ± 32.01^f^	1170.71 ± 26.85^f^
	BA1	1394.24 ± 34.24^a^	1376.72 ± 25.68^a^	1292.09 ± 21.23^a^	1214.06 ± 30.25^a^
	BA5	1376.72 ± 20.74^c^	1364.07 ± 29.85^c^	1278.41 ± 23.74^b^	1205.01 ± 27.77^c^
	BA10	1284.26 ± 31.21^d^	1264.12 ± 27.45^d^	1216.68 ± 21.05^e^	1194.22 ± 24.87^d^
**Chlorophyll *a* (mg kg**^**−**^**^1^)**	UW	682.27 ± 19.87^g^	672.91 ± 17.85^g^	614.07 ± 24.02^g^	486.76 ± 24.85^g^
	TW1	850.63 ± 19.02^d^	807.10 ± 15.22^d^	755.13 ± 20.31^d^	747.97 ± 12.73^d^
	TW5	743.41 ± 15.62^e^	724.94 ± 15.85^e^	714.69 ± 13.44^e^	607.77 ± 19.65^e^
	TW10	715.04 ± 10.28^f^	711.72 ± 11.20^f^	617.53 ± 25.62^f^	578.03 ± 25.65^f^
	BA1	984.43 ± 13.25^a^	957.90 ± 10.23^a^	893.19 ± 10.74^a^	839.16 ± 18.74^a^
	BA5	970.16 ± 12.33^b^	946.35 ± 16.20^b^	884.45 ± 26.38^b^	811.13 ± 20.14^b^
	BA10	910.70 ± 12.47^c^	834.07 ± 13.96^c^	808.25 ± 24.51^c^	763.80 ± 29.30^c^
**Chlorophyll *b* (mg kg**^**−**^**^1^)**	UW	469.26 ± 19.85^g^	417.43 ± 16.74^g^	414.25 ± 20.14^g^	390.68 ± 23.39^g^
	TW1	630.73 ± 12.57^d^	600.46 ± 26.39^d^	593.08 ± 23.44^d^	532.35 ± 24.98^d^
	TW5	573.35 ± 10.54^e^	570.03 ± 18.54^e^	540.19 ± 17.65^e^	510.94 ± 20.07^e^
	TW10	548.29 ± 11.24^f^	540.07 ± 16.20^f^	490.54 ± 23.41^f^	409.57 ± 20.02^f^
	BA1	646.12 ± 20.23^a^	631.73 ± 27.85^a^	606.89 ± 20.21^a^	593.61 ± 22.32^a^
	BA5	642.39 ± 13.20^b^	621.06 ± 13.65^b^	604.90 ± 15.62^b^	583.27 ± 21.55^b^
	BA10	636.02 ± 16.57^c^	606.71 ± 21.54^c^	603.99 ± 11.03^c^	574.68 ± 28.65^c^
**Ascorbic acid content (mg kg**^**−**^**^1^)**	UW	190.52 ± 19.98^g^	176.82 ± 21.01^g^	170.70 ± 27.11^g^	148.01 ± 12.98^g^
	TW1	369.62 ± 16.74^d^	216.59 ± 20.89^d^	197.37 ± 26.60^d^	167.76 ± 19.97^d^
	TW5	203.86 ± 18.21^e^	194.79 ± 23.50^e^	174.90 ± 19.01^e^	154.01 ± 16.84^e^
	TW10	197.82 ± 10.25^f^	183.36 ± 20.68^f^	172.02 ± 12.65^f^	153.61 ± 12.36^f^
	BA1	579.91 ± 21.36^a^	327.06 ± 23.65^a^	270.36 ± 20.65^a^	264.72 ± 11.37^a^
	BA5	547.82 ± 26.65^b^	306.39 ± 27.05^b^	251.51 ± 17.85^b^	225.61 ± 10.65^b^
	BA10	466.46 ± 20.34^c^	261.43 ± 22.21^c^	248.61 ± 12.98^c^	217.65 ± 14.55^c^
**Residual boron content (mg kg**^**−**^**^1^)**	BA1	2539.91 ± 20.24^c^	1914.86 ± 11.25^c^	1595.19 ± 18.24^c^	1252.49 ± 13.62^c^
	BA5	2552.42 ± 10.98^b^	2021.83 ± 17.84^b^	1703.48 ± 10.77^b^	1291.76 ± 10.97^b^
	BA10	2556.66 ± 17.25^a^	2304.22 ± 24.01^a^	1899.60 ± 12.41^a^	1391.39 ± 18.99^a^

a Different letters in the same column (for each analysis) show differences between sample groups according to the Duncan test (*P* < *0.05*).

b UW: unwashed, TW: tap water, BA: 1.0 % boric acid solution, 1, 5, and 10 indicate treatment times as min.

Papachristodoulou et al. [44] stated that the phenolic content during storage was higher in spinach washed with ozonated water than in untreated leaves. Although it has been reported that antioxidant phytochemicals can be degraded by cutting tissues, contrary findings are also found in the literature due to the simultaneous accumulation of phenolic compounds induced by cutting [2].

In the washing process using TW and BA, we observed that increasing the washing time decreased antioxidant activity and total phenolic content. Specifically, when comparing the samples at the beginning of storage, the antioxidant activity decreased from 61.32 mg kg^−1^ to 50.72 mg kg^−1^ in the TW samples and from 65.42 mg kg^−1^ to 58.48 mg kg^−1^ in the BA samples as the washing period increased from 1 to 10 min. Similarly, the total phenolic content decreased from 1384.36 mg kg^−1^ to 1204.31 mg kg^−1^ in the TW samples and from 1394.24 mg kg^−1^ to 1284.26 mg kg^−1^ in the BA samples. This finding can be attributed to antioxidant compounds that are water-soluble and can dissolve in washing water. Consequently, during extended washing times, a significant portion of the antioxidant compounds may remain in the water rather than the washed product. As a result, the product's antioxidant activity losses were found to be higher with prolonged washing times. This is in line with the studies conducted by Amin et al. [45] on spinach, where they reported that most phenolic compounds are transferred to the water during long-term boiling processes. It has been suggested that boiling spinach for less than 1 min helps preserve a higher antioxidant capacity. Similarly, Kuti and Konuru [46] also highlighted the water-soluble nature of the antioxidant activity in spinach. By considering the water-soluble characteristics of antioxidant compounds, our results indicate the importance of optimizing the washing time to minimize the loss of valuable antioxidant compounds during the processing of fresh-cut spinach.

The results showed a positive linear relationship between the total phenolic content and antioxidant capacity. A similar finding is from Howard et al. [47], which also stated that spinach flavonols have antioxidant properties. Cho et al. [43] reported that the hydroxyl groups of flavonoids and other phenolic compounds act as antioxidants with their free radical scavenging properties. Rodríguez‐Hidalgo et al. [38] attributed the total antioxidant activity in spinach leaves mainly to vitamin C. Similarly, Bottino et al. [2] also confirmed that the significant antioxidant capacity of spinach is due to the high amount of ascorbic acid and phenolic compounds, especially the flavonoids.

The results of the antioxidant activity analysis showed significant variations among different treatment groups throughout the storage period. The highest antioxidant activity values were observed in the samples treated with BA1, while the lowest was found in the UW samples. These differences were statistically significant (*P* < *0.05*). As the washing time increased from 1 to 10 min, there was a gradual decrease in antioxidant activity in both TW and BA samples. However, the antioxidant activity of BA samples remained consistently higher compared to TW samples at each time point. These differences were also statistically significant (*P* < *0.05*).

Regarding the total phenolic content, a similar trend was observed. The BA1 samples exhibited the highest values, while the UW samples had the lowest values. The total phenolic content decreased with increasing washing time in TW and BA samples. Statistically significant differences (*P* < *0.05*) were observed between different treatment groups at each time point, with BA samples consistently showing higher total phenolic content than TW samples. These findings suggest that the washing treatments, particularly the treatment of 1 % BA for 1 min, positively influence the antioxidant activity and total phenolic content of fresh-cut spinach during storage.

### 3.3 Chlorophyll *a* and *b* contents

Chlorophyll degradation is one of the leading quality loss factors in green-colored fresh-cut vegetables [48]. Decreases in chlorophyll content are often an essential indicator of the degree of cellular degradation or senescence [49]. Color change due to chlorophyll degradation is a normal process during vegetable ageing, and this phenomenon is reflected in the color parameters [44]. The green colour, an essential indicator of quality in leafy vegetables, is attributed to the chlorophyll pigments susceptible to spoilage, where degradation results from the enzymatic (chlorophyllase) conversion of chlorophyll *a* and *b* to pheophytin a and b, respectively [50]. Chlorophyll *a* and chlorophyll *b* content in all sample groups decreased during storage, and total chlorophyll degradation was higher in UW samples (Table 1). A similar finding was also reported by Hodges et al. [49]. However, Papachristodoulou et al. [44] observed that 38.5 %, 28.2 %, and 35.3 % of the initial chlorophyll *a*, *b*, and total chlorophyll content, respectively, were lost in untreated spinach after 12 days of storage at 8 °C. Generally, chlorophyll *a* was lost faster than chlorophyll *b* in all samples. While chlorophyll *a* was 682.27 mg kg^−1^ at the beginning of storage in the UW samples, it decreased to 486.76 mg kg^−1^ at the end of storage, and chlorophyll *b* decreased from 469.26 mg kg^−1^ at the beginning of storage to 390.68 mg kg^−1^ at the end of storage. These values are the lowest results obtained during storage. However, the highest amounts of chlorophyll *a* and *b* were observed in BA1 samples at the end of storage (839.16 and 593.61 mg kg^−1^, respectively). In the samples treated with TW, the amounts of chlorophyll *a* and *b* at the end of storage were determined in the 409.57–747.97 mg kg^−1^ range.

Similarly, Rodriguez-Hidalgo et al. [38] reported 517–547 mg kg^−1^ total chlorophyll in fresh-cut spinach washed with tap water and stored. Papachristodoulou et al. [44], on the other hand, reported that chlorophyll *a* and *b* losses were 38.5 % and 28.2 %, respectively, in untreated spinach at the end of the 12-day storage, while it was 13.9 % and 6.5 %, respectively, in spinach washed with ozonated water. The increase in washing time in TW and BA treatments negatively affected the chlorophyll content, similar to the antioxidant activity and total phenolic content. In the samples treated with TW, 747.97 mg kg^−1^ chlorophyll *a* and 532.35 mg kg^−1^ chlorophyll *b* were obtained at the end of storage with 1 min washing, while these values decreased to 578.03 and 409.57 mg kg^−1^, respectively, after 10 min of washing. Similarly, 839.16 and 593.61 mg kg^−1^ chlorophyll *a* and *b* contents were obtained in the samples treated with BA at the end of the storage, with 1 min of washing, while these values decreased to 763.80 and 574.68 mg kg^−1^, respectively, with 10 min of washing. Chlorophyll contents were maximally retained in BA1 samples (86 %–91 %), which was evaluated as the most effective method. This indicates that BA solution can deactivate the chlorophyllase enzyme (optimal pH 8.5), which is involved in chlorophyll degradation by creating acidic conditions [44,51]. It has also been reported that low-pH solutions effectively reduce pigment loss [52]. However, the increase in washing time with BA increased the chlorophyll loss, which can be explained by the leaching of the components into the washing solution [53].

**Fig. 2:**
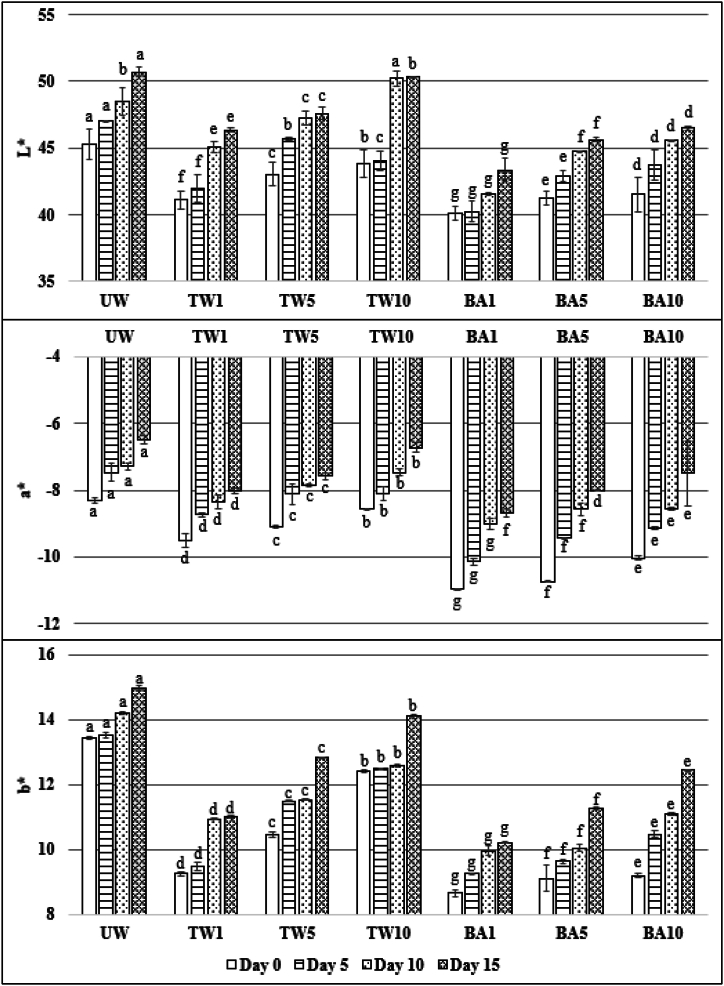
Effects of storage time on the color (L*: (100) white, (0) black; a*: (+)red, (−)green; b*: (+)yellow, (−)blue) of unwashed (UW), tap water (TW), and boric acid (BA) washed spinach samples at 1, 5, and 10 min of washing times. (^a^ Different letters used in the columns of each storage day indicate significant differences (*P* < *0.05*) between each treatment according to the Duncan's test.).

Additionally, as reported by Rodríguez‐Hidalgo et al. [38], differences in chlorophyll *a* and *b* loss between sample groups were also reflected in the color values (Fig. 2). Chlorophyll *a* is bright blue-green, while chlorophyll *b* is more yellow-green. The decrease in chlorophyll content during storage also caused the color values to change from green to yellow. The effectiveness of BA treatments in controlling the yellowing of fresh-cut spinach is associated with its strong oxidation potential, which makes the environment acidic. However, ascorbic acid, an essential antioxidant in spinach, can also inhibit chlorophyll degradation reactions [54], so maximum chlorophyll preservation in BA1 treatment is also supported by our ascorbic acid results (Table 1).

The chlorophyll *a* and *b* content analysis revealed significant differences among the treatment groups during the storage period. At the beginning of storage, the UW samples exhibited the highest chlorophyll *a* and chlorophyll *b* content, while the BA1 samples had the highest content at the end of storage. These differences were statistically significant (*P* < *0.05*). During the storage period, chlorophyll *a* and chlorophyll *b* content decreased in all sample groups. The UW samples consistently showed the lowest chlorophyll content, while the BA1 samples had the highest values. The differences in chlorophyll content between the UW and BA1 samples were statistically significant at each time point (*P* < *0.05*). Increasing the washing time in TW and BA treatments negatively affected the chlorophyll content, similar to the antioxidant activity and total phenolic content. The longer washing times they resulted in a gradual decrease in chlorophyll *a* and chlorophyll *b* content. However, the preservation of chlorophyll was maximized in the BA1 samples, indicating the effectiveness of the 1 % boric acid treatment in preserving chlorophyll content during storage. The differences in chlorophyll content between different treatment groups were found to be statistically significant (*P* < *0.05*) at each time point. These findings suggest that applying 1 % BA for 1 min is the most effective treatment for preserving chlorophyll content in fresh-cut spinach during storage.

### 3.4 Ascorbic acid content

Ascorbic acid is a critical component that acts as an antioxidant in vegetables by inhibiting the reactions that disrupt chlorophyll, and it acts as a radical scavenger during oxidative stress [54]. Ascorbic acid is reversibly oxidized to dehydroascorbic acid in plant tissues, and the natural balance in spinach leaves is maintained by high ascorbic acid and low dehydroascorbic acid concentrations [41]. Reyes et al. [30] emphasized that reduced ascorbic acid is found at higher levels in cut fruit and vegetable tissues. Ascorbic acid protects against enzymatic browning mainly by controlling the polyphenol oxidase activity and is widely used in various products in the food industry for this purpose [2].

The ascorbic acid content decreased in all sample groups during storage (Table 1). The maximum reduction was observed in UW samples during storage, and our data are similar to the literature. Cocetta et al. [55] reported that the ascorbic acid content of fresh-cut spinach stored in the cold showed significant changes from the third day (52 % reduction) and decreased (68 % reduction) during storage. Bottino et al. [2] reported that the ascorbic acid content of fresh-cut spinach decreased over time in cold storage.

However, researchers emphasized that although vitamin C decreases over time in fresh-cut spinach, its nutritional values are preserved due to other antioxidant compounds. A study by Kaur et al. [54] found that the ascorbic acid content in fresh-cut spinach decreased to 80–120 mg kg^−1^ at the end of storage. In the study by Fan and Sokorai [56], it was determined that ascorbic acid decreased from 6450 mg kg^−1^ to 4320 mg kg^−1^ at the end of storage. At the beginning and end of storage, the lowest ascorbic acid content was observed in UW samples (190.52 and 148.01 mg kg^−1^, respectively) and the highest in BA1 samples (579.91 and 264.72 mg kg^−1^, respectively). Similar to the antioxidant activity, total phenolic, chlorophyll *a*, and *b* content, ascorbic acid content decreased with increasing washing time in TW and BA treatments. Based on the beginning of storage, the content of ascorbic acid, which was 369.62 mg kg^−1^ in 1 min washing with TW, decreased to 197.82 mg kg^−1^ when this period was 10 min.

Similarly, the content of ascorbic acid, which was 579.91 mg kg^−1^ in 1 min of washing with BA, decreased to 466.46 mg kg^−1^ after 10 min of washing. At the end of storage, the ascorbic acid content (217.65–264.72 mg kg^−1^) in BA treatments was more stable and higher than TW (153.61–167.76 mg kg^−1^). When BA treatment is evaluated in itself, the BA1 process gives the best results in terms of protection of ascorbic acid. Reducing the pH to acidic conditions using BA may have stabilized the ascorbic acid content [52]. When evaluated in general, the decrease in ascorbic acid content is the main factor responsible for the decrease in antioxidant capacity, and these two cases show parallel results (Table 1). Ascorbic acid in spinach also prevented enzymatic browning by reducing polyphenol oxidase enzyme reactions, which agrees with the color results (Fig. 2).

The analysis of ascorbic acid content demonstrated significant differences among the treatment groups throughout the storage period. At the beginning of storage, the UW samples had the lowest ascorbic acid content, while the BA1 samples exhibited the highest. These differences were statistically significant (*P* < *0.05*). During the storage period, ascorbic acid content decreased in all sample groups, with the UW samples consistently showing the highest reduction. The BA1 samples consistently had the highest values at each time point, and the differences between the UW and BA1 samples were statistically significant (*P* < *0.05*) at each time point. Increasing the washing time in both TW and BA treatments resulted in a decrease in ascorbic acid content. The longer washing times have led to a gradual reduction in ascorbic acid content. However, the preservation of ascorbic acid was maximized in the BA1 samples, indicating the effectiveness of the 1 % BA treatment in preserving ascorbic acid content during storage. The differences in ascorbic acid content between different treatment groups were found to be statistically significant (*P* < *0.05*) at each time point. These findings suggest that applying 1 % BA for 1 min is the most effective treatment for preserving ascorbic acid content in fresh-cut spinach during storage.

### 3.5 Residual boron content

In the samples washed with BA, as the washing time increased, the residual boron content in the product increased, whereas the residual boron content in the washing solution decreased. During the storage, the residual boron content decreased in all sample groups, and the lowest residual boron was observed in the samples subjected to BA1 treatment (Table 1). In the solutions used after washing with BA, the residual boron amount was found to be 53.88, 48.76, and 42.74 mg kg^−1^ for BA1, BA5, and BA10, respectively. Longer contact time with BA solution resulted in higher BA penetration into the spinach leaves, and accordingly, its amount in the washing solution decreased. Residual boron amounts in the sample and the washing solution provided results related to each other. This relationship is explained by the fact that in the BA1 treatment, where the treatment duration is shorter, the residual boron amount in the sample is lower. At the same time, it is higher in the washing water. It is important to note that individuals are primarily exposed to boron components through their diet and drinking water. Exposure limits play a critical role in assessing the potential effects of these components, and it is worth mentioning that most boron compounds have low toxicity and do not pose significant health risks [7]. Hence, considering the lower boron content in the final product and the minimal exposure through diet and drinking water, the higher levels of boron in the washing water do not present a risk to individuals. It is crucial to note the significance of the residue levels in the washing water post-process, similar to the residues in the disinfectant solutions used for food washing.

The analysis of residual boron content revealed significant differences among the treatment groups at each time point. The residual boron content increased in the product as the washing time with BA solution increased, while it decreased in the washing solution. These differences were found to be statistically significant (*P* < *0.05*). During the storage period, the residual boron content decreased in all sample groups. The BA1 samples consistently had the lowest residual boron content at each time point, while the BA10 samples had the highest values. The differences in residual boron content between different treatment groups were found to be statistically significant (*P* < *0.05*) at each time point.

### 3.6 Moisture content

Weight loss is the most important issue in the storage of leafy vegetables [57]. Moisture content is an important indicator of quality loss in spinach, which spoils very quickly due to water loss [48]. Water loss is a significant cause of post-harvest spoilage as it leads to not only direct quantitative losses but also appearance and textural quality deterioration.

The moisture content of UW and TW samples decreased throughout the storage period, while an increase was observed in BA-treated samples (Table 1). Specifically, the initial moisture content of UW samples, starting at 89.19 %, decreased to 86.57 % by the end of storage. This indicates that the water in the vegetable tissue is gradually removed, leading to a decrease in moisture content per unit volume, which is an important quality indicator for vegetables to retain water in their structure. Similarly, Hodges et al. [49] also observed an increase in electrolyte leakage during the storage of fresh-cut spinach that was washed and rinsed with chlorine dioxide, indicating a decrease in moisture content due to water removal from the structure. Fan et al. [57] reported a decrease in fresh weight of spinach during storage. The moisture content increased from 90.23 % to 90.71 % at the beginning of storage and from 87.36 % to 88.81 % at the end of storage, with increasing time in the TW treatment (from 1 min to 10 min). The decrease in moisture content after TW treatment is also an indicator of water removal from the structure. However, in BA treatments, the opposite was observed: the moisture content was 88.69 % at the beginning of storage after 1 min of washing, decreased to 87.29 % after 10 min of washing, and was 89.81 % and 89.16 % at the end of storage, respectively. In other words, as the washing time increased, the moisture content decreased. The extended washing time with BA allowed for better penetration of BA into the product, thus retaining water in the structure. The highest moisture content was observed in the BA1 samples (89.81 %) at the end of storage. In this treatment, as the time increased, the water in the structure decreased due to damage to the leaf tissue caused by the acidic solution. However, the moisture content remained high in the BA1 samples because the water content in the fresh-cut spinach leaves was preserved.

The moisture content showed significant variations among the treatment groups at each time point. Throughout the storage period, the moisture content decreased in UW and TW samples, while an increase was observed in BA-treated samples. These differences were found to be statistically significant (*P* < *0.05*). In TW treatments, the moisture content increased with increasing washing time, reflecting the removal of water from the spinach structure. These differences in moisture content between different treatment groups were found to be statistically significant (*P* < *0.05*) at each time point. Conversely, in BA treatments, the moisture content showed an opposite trend. The differences in moisture content between different BA treatment groups were found to be statistically significant (*P* < *0.05*) at each time point.

As a result, BA treatments showed a potential in retaining water in the structure of fresh-cut spinach, as indicated by the moisture content results. These findings suggest that BA can create a barrier against moisture loss and help preserve the water content in the product.

### 3.7 Color

The color change is the first visible sign of deterioration that affects the economic value of leafy vegetables, and preserving the original color is crucial as it serves as an important quality indicator until the vegetables are consumed [58]. Color changes resulting from chlorophyll degradation are a natural process during the shelf life of green leafy vegetables, with the yellowing of spinach leaves being recognized as the most significant post-harvest change [44]. Paleness in color, a decrease in greenness, and an increase in yellowness were observed in the UW samples during storage (Fig. 2).

These findings were associated with an increase in the L* value ranging from 45.3 ± 1.1 to 50.71 ± 0.34, a* value ranging from −8.31 ± 0.07 to −6.49 ± 0.12, and b* value ranging from 13.44 ± 0.03 to 14.97 ± 0.08. The highest color change in UW samples was observed in the L* index (45.3–50.71). Higher L* values indicate the whiteness of the samples, while low L* values indicate darkness. During storage, the L* value increased as the color of the UW samples became lighter. However, while the -a* value, an indicator of greenness, decreased, the +b* value, an indicator of yellowness, increased. Short washing times (1 min) better maintained color values better in TW and BA samples. Based on the beginning of the storage, the L*, a* and b* values were 41.13 ± 0.66, −9.51 ± 0.20, and 9.24 ± 0.08, respectively, at the 1 min TW treatment, while these values increased to 43.82 ± 1.04, −8.57 ± 0.03, and 12.41 ± 0.04, respectively, at the 10 min treatment. Similarly, while these values were 40.11 ± 0.54, −10.97 ± 0.01, and 8.64 ± 0.11, respectively, in the 1 min BA treatment, they increased to 41.52 ± 0.10, −10.03 ± 0.11, and 9.18 ± 0.03, respectively, in the 10 min BA treatment. Extended washing of the leaves had negative effects on the color. At the end of storage, the lowest L*, a*, and b* values were determined in the BA1 treatment (40.11, −10.97, and 8.64, respectively). The statistical analysis of the color results revealed significant differences between all treatment groups for L*, a*, and b* values at each storage day (*P* < *0.05*). These findings indicate that the washing treatments significantly influenced the color values of the spinach samples throughout the storage period. Each treatment had different superscript letters indicating significant variation (Fig. 2). These findings emphasize the impact of different washing methods on the color values of the spinach samples. The results indicate that prolonged washing times had negative effects on color, resulting in decreased lighter color and increased yellowness. Overall, the statistical analysis confirms the conclusion that the washing treatments significantly influenced the color values of the spinach samples during storage. Changes in colour values, especially greens, correlate with chlorophyll results (Table 1). Consistent with our results, Poimenidou et al. [59] determined L*, a*, and b* values in unwashed spinach as 36.9, −15.0, and 19.5, respectively. Finten et al. [60], on the other hand, stated that L*, a*, and b* values increased during storage in spinach washed with sodium hypochlorite, and citric acid solutions. Papachristodoulou et al. [44] also observed that the yellowness decreased in spinach that was washed with ozonated water before packaging. Our study showed that washing with BA demonstrated a similar level of effectiveness to that with ozone in terms of color preservation.

### 3.8 Texture properties

The leaf tissue is an important parameter in spinach quality, resulting in the deterioration of the cell walls over time; water and solids are released into the intercellular space and cause tissue loss [61]. In the texture analysis of spinach leaves, force and distance represent the maximum shear force and work required to cut samples, respectively. Softening in spinach results from the breakdown of cellular components [56].

It has been reported in the literature that the physical parameters related to texture in vegetables are difficult to analyze and that these properties can vary greatly depending on the raw material [61], so the results obtained from this study were evaluated on their own (based on differences between treatments). While the texture properties of UW and TW samples weakened during storage, BA samples, on the other hand, exhibited improved texture properties (Table 2). In UW samples, puncture strength from 1.60 N at the beginning of storage decreased to 0.98 N at the end of storage, and the puncture distance from 50.02 mm to 30.97 mm at the end of storage.

**Table 2:** Changes in the texture characteristics of the sample groups during the storage periods.

	Sample^b^	Day 0	Day 5	Day 10	Day 15
**Puncture strength (N)**	UW	1.60 ± 0.18^a^	1.24 ± 0.09^g^	1.02 ± 0.07^g^	0.98 ± 0.03^g^
	TW1	1.49 ± 0.13^c^	1.44 ± 0.27^d^	1.41 ± 0.05^d^	1.30 ± 0.05^d^
	TW5	1.44 ± 0.03^e^	1.42 ± 0.33^e^	1.28 ± 0.02^e^	1.20 ± 0.47^e^
	TW10	1.40 ± 0.15^f^	1.39 ± 0.07^f^	1.13 ± 0.12^f^	1.04 ± 0.06^f^
	BA1	1.58 ± 0.22^b^	1.65 ± 0.21^a^	1.74 ± 0.08^a^	1.81 ± 0.14^a^
	BA5	1.49 ± 0.13^c^	1.61 ± 0.18^b^	1.66 ± 0.11^b^	1.73 ± 0.20^b^
	BA10	1.46 ± 0.42^d^	1.54 ± 0.10^c^	1.58 ± 0.16^c^	1.61 ± 0.10^c^
**Puncture distance (mm)**	UW	50.02 ± 0.09^a^	46.84 ± 0.17^g^	37.71 ± 0.08^g^	30.87 ± 0.05^g^
	TW1	49.57 ± 0.12^b^	49.29 ± 0.16^d^	40.82 ± 0.04^f^	39.56 ± 0.19^d^
	TW5	48.84 ± 0.10^e^	48.52± 0.11^e^	42.55 ± 0.01^d^	37.65 ± 0.12^e^
	TW10	48.51 ± 0.14^f^	48.35 ± 0.08^f^	41.45 ± 0.10^e^	37.40 ± 0.14^f^
	BA1	49.53 ± 0.20^c^	51.64 ± 0.10^a^	52.14 ± 0.05^a^	52.78 ± 0.04^a^
	BA5	49.45 ± 0.18^d^	50.27 ± 0.15^b^	52.03 ± 0.07^b^	51.99 ± 0.15^b^
	BA10	49.44 ± 0.16^d^	50.02 ± 0.07^c^	51.44 ± 0.12^c^	51.72 ± 0.06^c^

a Different letters in the same column (for each analysis) show differences between sample groups according to the Duncan test (*P* < *0.05*).

b UW: unwashed, TW: tap water, BA: 1.0 % boric acid solution, 1, 5, and 10 indicate treatment times as min.

Tsironi et al. [62] found puncture strength values as 1.23–1.75 N for different types of lettuce, which is similar to our data. Sánchez et al. [61] also reported the mean maximum puncture force value in spinach as 1.93 N. The lowest texture quality throughout the process belonged to these samples. Nguyen et al. [63] also reported a significant decrease in the texture of spinach after 10 days of storage. Prolonged application in TW and BA treatments had a negative effect on tissue properties. While the puncture strength was 1.30 and 1.81 N, respectively, in 1 min of TW and BA treatment at the end of storage, these values decreased to 1.04 and 1.61 N, respectively, after 10 min of treatment.

Similarly, the puncture distance value decreased from 39.56 to 52.78 mm to 37.40 and 51.72 mm, respectively. In the comparison of TW and BA treatment, the texture of TW samples worsened during storage, while the texture of BA samples improved. These results indicate that BA has a tissue protective effect on the product over time. The statistical analysis of the puncture strength and puncture distance values showed significant differences between the treatment groups on each storage day (*P* < *0.05*). These findings indicate that the washing treatments significantly influenced the texture properties of the spinach samples. The chemical content was better preserved in products treated with an acidic solution by the use of BA (Table 1), which may have played a role in the preservation of the texture.

Additionally, the texture properties are also related to the water in the structure of the spinach leaf, and these results are consistent with the content of moisture content obtained during storage. Furthermore, sensory evaluation results (see Section 3.10), results were obtained to support this phenomenon. Babic and Watada [64] also reported that surface acidity affects shear force in fresh-cut spinach. In conclusion, the BA1 treatment increases the strength of the leaves and protects the tissue better.

### 3.9 SEM images

**Fig. 3:**
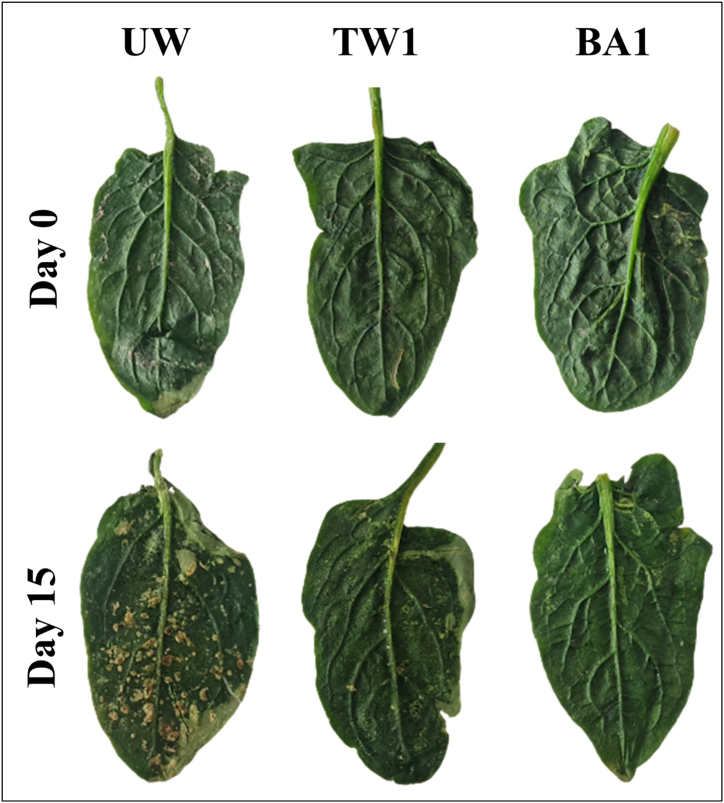
Images of the unwashed (UW), washed for 1 min with tap water (TW1), and washed for 1 min with a 1.0 % boric acid solution (BA1) of the spinach leaves at the initial and final storage days.

**Fig. 4:**
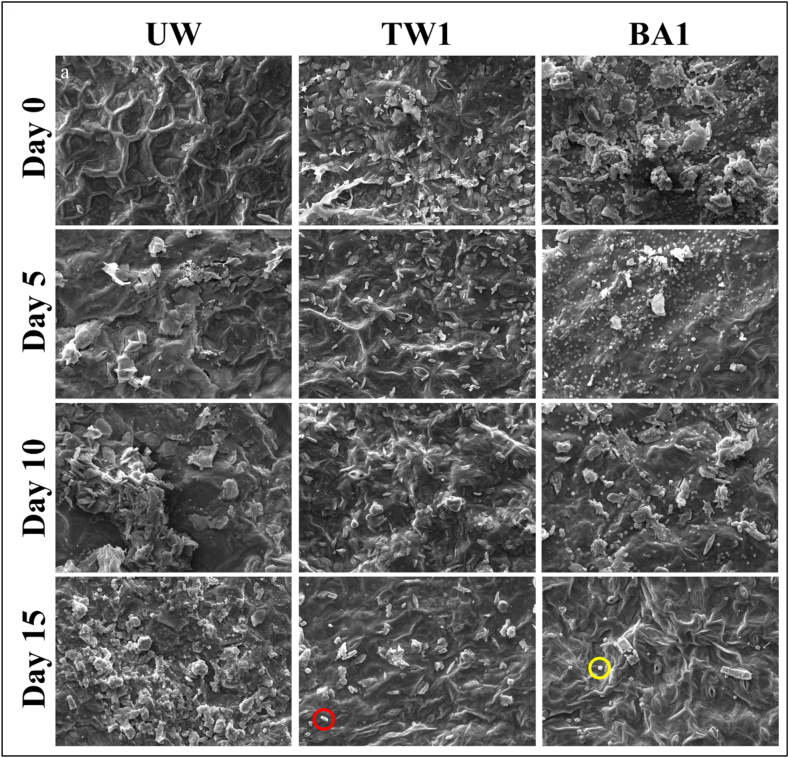
SEM images of the unwashed (UW), washed for 1 min with tap water (TW1), and washed for 1 min with a 1.0 % boric acid solution (BA1) of the spinach leaves at different storage days (Chlorine residue is indicated by a red circle on the TW1 sample at day 15, while boric acid crystal residue is marked by a yellow circle on the BA1 sample at day 15).

The microstructure of spinach leaves serves as an indicator of tissue damage [65]. The SEM analysis was performed on UW and various washing samples, with TW1 and BA1 samples yielding the best results within their respective treatment groups. Fig. 3 presents fresh images of these samples for both the initial and final storage days, while Fig. 4 provides SEM images of these samples for each storage period. The results of the SEM analysis clearly depict the changes in the fresh leaves shown here.

At the beginning, the integrity of the cell and the intact structure of the leaf veins can be observed, while on the fifth day of storage, the cell surface is damaged. In the further storage processes, tissue integrity is observed, but it is completely lost, and fragmentation occurs.

When SEM images of the TW1 sample were examined at the beginning of storage, chlorine residues originating from tap water were visible on the product surface, which is relatively more whole. In the following days of storage, the visibility of chlorine residue decreased, and the tissue stroma was also damaged. On the last day of storage, there is no evidence of the stoma structure in the image and it is seen that the chlorine residue has decreased a lot. There was a loss of turgor, and damage to the stoma occurred due to the serious structural surface damage, such as cracks and dehydration observed in UW and TW samples.

SEM images of BA1 samples reveal a distinctive feature. In contrast to the rod-like appearance of the chlorine residue, the BA residue appears more circular and uniform. This finding agrees with the powder's chemical structure. Chlorine, a chemical element with strong oxidizing properties, tends to form compounds that adhere to surfaces in irregular shapes. In contrast, boric acid appears to form in a more spherical and orderly manner. At the beginning of the storage, BA residue is seen on the sample surface in small sizes but in large quantities, the structure can be evaluated as a whole and intact. As the storage days progressed, notable reductions in BA residue were detected, but it can be said that tissue integrity is still preserved. On the last day of storage, it is seen that the stoma is still intact, the vascular structures are evident, and the tissue integrity is in excellent condition.

In the SEM images of UW, TW1, and BA1 samples obtained during storage, the results indicated that the microstructure in spinach was best preserved by BA1 treatment. This finding is consistent with the chemical composition (especially moisture content) and texture properties of the sample. While there were obvious structural differences in BA1 samples, Koseki et al. [66] and Rico et al. [67] did not observe any significant structural differences between washing with electrolyzed water and chlorinated water fresh-cut lettuce. Also, our SEM images of fresh spinach differ significantly from those of Bilbao-Sainz et al. [65], which may be mainly due to differences in raw material or method/device.

### 3.10 Sensory evaluation

**Fig. 5:**
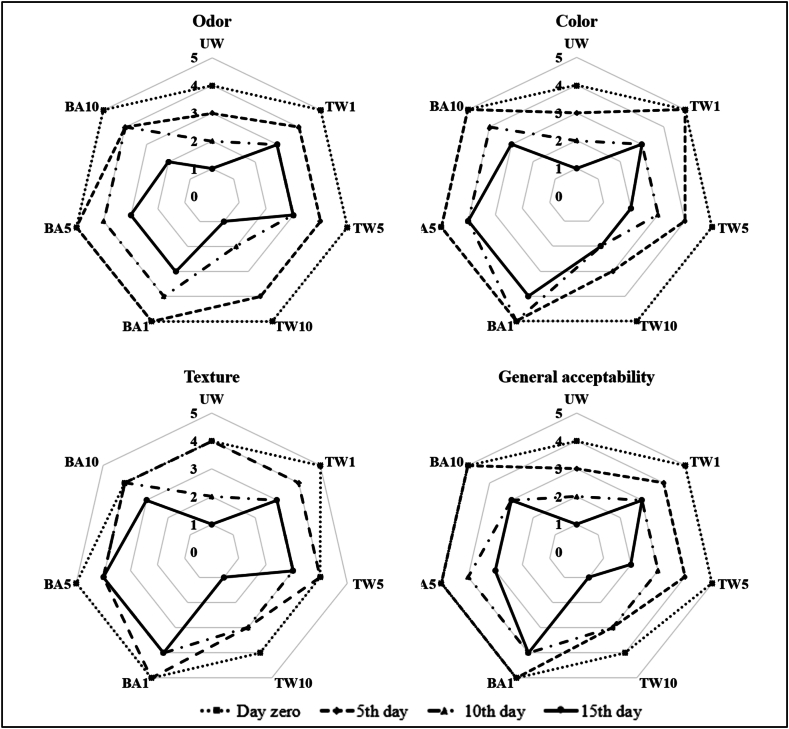
Sensory evaluation results of sample groups (UW: unwashed, TW: tap water, BA: 1.0 % boric acid solution, 1, 5, and 10 indicate treatment times as min).

Freshness is the key quality factor desired for leafy green vegetables, and the intensity, clarity, and uniformity of green are particularly desired. In all sample groups, the sensory scores of the samples continued to decrease during storage (Fig. 5).

On the first day of storage odor score had only 4 points for the UW sample and 5 points for the others (TW and BA samples). Because of the washing treatment, most raw odor gets transferred into the washing water; thus, washed spinach receives higher sensory scores. While the odor scoring decreased to 3 points in UW samples on the third day of storage, indicating that they were no longer marketable, it took ten and fifteen days to reach to this score in the TW and BA samples, respectively. The green color, which is associated with freshness in spinach, was evaluated with only 4 points in the UW samples and 5 points in other samples on the first day of storage. While UW samples were evaluated as unmarketable (3 points) as of the fifth day, similar decreases were observed on the tenth day in TW samples. In BA samples, only BA10 samples were evaluated with 3 points on the last day of storage, while BA1 and BA5 samples were evaluated with 4 points even on the last day.

The sensory evaluation of the color is also similar to the results of the color analysis, as seen in Fig. 2. The panellists negatively evaluated the change of colour in fresh leaves from green to yellow (decrease in -a* value and increase in +b* value). The texture of the spinach leaves, as well as the smell and colour, is an important phenomenon that governs consumer perception and affects the consumer's perception of freshness.

When examining the texture evaluation by the panellists, it was observed that they preferred a strong, thick, uniform, and intact structure in spinach. Texture evaluation was conducted on UW samples and samples washed with TW and BA for an extended duration (10 min), resulting in a 4-point evaluation on the first day of storage. Unlike other sensory parameters, TW10 samples were evaluated with a score of 3 on the third day of storage, possibly due to the damage to product texture by prolonged tap water washing. On the tenth day, UW samples were evaluated with 2 points, TW samples were evaluated with 3 points, and these 2 groups were determined as unmarketable. When BA samples were evaluated within themselves, only BA10 samples were evaluated with 3 points at the end of storage. BA1 and BA5 samples were always evaluated with 4–5 points throughout storage in terms of texture. The sensory evaluation scores are in line with the texture analysis results in Table 2. Panellists evaluated the overall acceptability potential of the sample groups by considering the perceptions of smell, colour, and texture together. While UW samples were assessed starting from the third day, TW and BA10 samples received rejection scores only from the tenth day. BA5 samples were determined as unmarketable by the panellists at the end of storage. The overall acceptance score of only BA1 samples was never evaluated below 4 points among all sample groups during storage. Considering these results, it was determined that while the samples washed with tap water suffered a loss of quality rapidly, the short-term (1 min) treatment in the BA washing treatment protected the sensory quality criteria of the product better.

The results of the sensory evaluation are compatible with similar research in the literature and physicochemical analysis results. Dewhirst et al. [68] also reported that the visual quality of spinach washed with chlorinated water and spring water decreased during storage. In terms of general acceptability, the panellists considered the UW samples as non-marketable from day 4, TW samples from day 9, and BA10 samples from day 14, while they did not evaluate the BA1 and BA5 samples with a score at the non-consumable limit. Nguyen et al. [63] reported that the sensory qualities of spinach leaves washed with chlorine dioxide decreased significantly from day 7. Similarly, Papachristodoulou et al. [44] indicated that at the end of the storage period, spinach that was not treated was considered unacceptable, while spinach washed with ozonated water was considered acceptable.

## 4 Conclusions

Fresh-cut spinach undergoes minimal processing before reaching the market, typically with a shelf life of 5–6 days. However, such processing methods can inadvertently lead to tissue damage, hastening the deterioration of nutritional and overall quality properties. Among these, the washing process stands out as pivotal. Presently, chlorine and its alternatives are widely utilized for decontaminating fresh-cut vegetables, each with its own set of advantages and limitations. Ensuring microbial stability, color retention, tissue firmness, and nutritional integrity throughout the shelf life of vegetables is paramount, and any applied solution for decontamination should not compromise sensory perceptions.

In this study, we evaluated the efficacy of washing fresh-cut spinach with BA solutions in maintaining both microbiological safety and physicochemical quality parameters. Our findings suggest that optimal results are achieved with a 1.0 % (w/v) boric acid solution for 1 min. Shortening the washing duration with a boric acid solution proved more effective in preserving quality parameters and extending shelf life, encompassing physical, chemical, and sensory aspects. The adoption of a boron-based disinfectant presents a sustainable, environmentally friendly alternative likely to resonate with both producers and consumers.

Despite the significant insights gleaned from our study, it is crucial to acknowledge several limitations that may influence the interpretation and generalization of our results. Firstly, the utilization of boric acid for fresh-cut spinach decontamination is a relatively novel approach, necessitating a deeper understanding of its chemical reactions during storage to interpret its effects accurately. Future research efforts should delve into underlying mechanisms of action and cause-and-effect relationships regarding their impact on quality parameters. Additionally, while our study focused on short-term effects, the long-term implications of boric acid washing remain unclear. Investigating extended storage periods would offer a more comprehensive understanding of its practical applicability.

Moreover, the inherent variability in spinach varieties and growing conditions represents an important limitation, potentially affecting the efficacy of boric acid washing. Furthermore, while sensory evaluation is widely used, its subjectivity and susceptibility to various influences warrant consideration. Employing more objective methods or diversifying the consumer base could enhance the reliability of sensory findings.

Lastly, while boric acid is generally regarded as environmentally friendly, further investigation into its potential environmental impact is necessary to ensure sustainable practices.

In light of these limitations, future research endeavours should aim to address these challenges, providing comprehensive insights into the practical application and implications of boric acid washing in the fresh-cut produce industry. Additionally, it is essential for manufacturers and stakeholders to assess the feasibility of adopting boric acid washing as a sustainable and effective decontamination method, considering factors such as scalability, cost-effectiveness, and regulatory considerations.

## Funding statement

The research leading to these results received funding from 10.13039/100007613Ankara University Scientific Research Projects (BAP) under Grant Agreement No 21B0443003.

## Lead contact

Angelo Maria Giuffrè (email: amgiuffre@unirc.it)

## Data availability

Data will be made available on request.

## CRediT authorship contribution statement

**Bahar Demircan:** Writing – original draft, Visualization, Validation, Software, Methodology, Investigation, Formal analysis, Data curation. **Yakup Sedat Velioglu:** Writing – review & editing, Supervision, Resources, Project administration, Methodology, Investigation, Funding acquisition, Conceptualization. **Angelo Maria Giuffrè:** Writing – review & editing, Visualization, Supervision, Software, Resources, Project administration, Investigation, Data curation, Conceptualization.

## Declaration of competing interest

The authors declare that they have no known competing financial interests or personal relationships that could have appeared to influence the work reported in this paper.
